# Catalytic-CO_2_-Desorption Studies of BZA-AEP Mixed Absorbent by the Lewis Acid Catalyst CeO_2_-γ-Al_2_O_3_

**DOI:** 10.3390/molecules28114438

**Published:** 2023-05-30

**Authors:** Shenghua Liu, Xudong Mao, Hao Chen, Xinbo Zhu, Guohua Yang

**Affiliations:** Faculty of Maritime and Transportation, Ningbo University, Ningbo 315832, China; 2011084032@nbu.edu.cn (S.L.); 2011084034@nbu.edu.cn (X.M.); 2211120024@nbu.edu.cn (H.C.); zhuxinbo@nbu.edu.cn (X.Z.)

**Keywords:** carbon capture, BZA-AEP absorbent, CeO_2_-γ-Al_2_O_3_ catalyst, catalytic desorption, desorption mechanism

## Abstract

Traditional organic amines exhibit inferior desorption performance and high regeneration energy consumption. The implementation of solid acid catalysts presents an efficacious approach to mitigate regeneration energy consumption. Thus, investigating high-performance solid acid catalysts holds paramount importance for the advancement and implementation of carbon capture technology. This study synthesized two Lewis acid catalysts via an ultrasonic-assisted precipitation method. A comparative analysis of the catalytic desorption properties was conducted, encompassing these two Lewis acid catalysts and three precursor catalysts. The results demonstrated that the CeO_2_-γ-Al_2_O_3_ catalyst demonstrated superior catalytic desorption performance. Within the desorption temperature range of 90 to 110 °C, the average desorption rate of BZA-AEP catalyzed by the CeO_2_-γ-Al_2_O_3_ catalyst was 87 to 354% greater compared to the desorption rate in the absence of the catalyst, and the desorption temperature can be reduced by approximately 10 °C. A comprehensive analysis of the catalytic desorption mechanism of the CeO_2_-γ-Al_2_O_3_ catalyst was conducted, and indicated that the synergistic effect of CeO_2_-γ-Al_2_O_3_ conferred a potent catalytic influence throughout the entire desorption process, spanning from the rich solution to the lean solution.

## 1. Introduction

Elevated emissions of greenhouse gases are contributing to severe climate change. The international community has reached a consensus on the necessity of controlling greenhouse gas emissions. According to the IPCC’s 1.5 °C global warming report [1], human activities are estimated to have induced approximately 1.0 °C of global warming above pre-industrial levels, with a possible range of 0.8 °C to 1.2 °C. Should the current rate of warming persist, global warming may reach 1.5 °C between 2030 and 2052. Carbon capture and storage (CCS) represents a crucial means of reducing carbon dioxide emissions in the future, with the most promising application being CO_2_ capture by organic amines [2]. However, traditional organic amine absorbents exhibit the drawback of high regeneration energy consumption. Introducing catalysts can facilitate carbamate decomposition and CO_2_ desorption at reduced temperatures [3].

Idem et al. [4] first reported the employment of solid acid catalysts in the CO_2_ desorption process involving amine-rich solutions. The researchers demonstrated that H-ZSM-5 and γ-Al_2_O_3_, two prevalent industrial solid acid catalysts, possess the ability to enhance the desorption performance of mono-ethanolamine (MEA) solution. Shi et al. [5] employed single and mixed amines (MEA, MEA-MDEA (N-methyl-diethanolamine), MEA-DEAB (4-(diethylamino)-2-butanol)) to compare the catalytic desorption effects of γ-Al_2_O_3_ and H-ZSM-5 at 90–95 °C. They discovered that the catalytic efficacy of H-ZSM-5 surpassed that of γ-Al_2_O_3_. The addition of MDEA or DEAB (as a tertiary amine) to MEA provides R_1_R_2_R_3_N and HCO_3_^−^, splitting the free energy gap and reducing it. In the CO_2_ lean solution region, γ-Al_2_O_3_ (Lewis acid) is more effective, replacing the role of HCO_3_^−^. The role of HCO_3_^−^ in the CO_2_ lean solution region is negligible, while H-ZSM-5 (Brønsted acid) is effective throughout the load range by providing protons. Any free proton available will attack the carbamate N atom. From the perspective of the optimal molecular structure, protons attach to the N atom, transform the sp2 hybridization of N and C atoms into sp3 hybridization, break the delocalized N-COO- conjugation, and ultimately stretch the N-C bond to prepare for the N-C bond breaking [4]. Liang et al. [3] compared the catalytic desorption effects of γ-Al_2_O_3_, H-ZSM-5, and H-Y on MEA solutions. The results revealed that, in the CO_2_ lean solution region, the catalyst performance (desorption rate) order was: γ-Al_2_O_3_ > H-ZSM-5 > H-Y. γ-Al_2_O_3_ promotes CO_2_ desorption through two primary effects. Firstly, γ-Al_2_O_3_ can attack carbamate to release CO_2_. Secondly, γ-Al_2_O_3_ can facilitate the deprotonation of protonated amines in the lean solution region. Transition metal oxides can promote the decomposition of carbamates. Bhatti et al. [6] investigated the performance of five distinct transition metal oxide catalysts, V_2_O_5_, MoO_3_, WO_3_, TiO_2_, and Cr_2_O_3_, examining their effects on the regeneration of amine solutions in the temperature range of 35–86 °C. The results demonstrated that the regeneration performance of MoO_3_ significantly outperformed the non-catalytic amine regeneration system, being nearly twice as effective as the MEA solvent. Additionally, the other catalysts within this temperature range exhibited substantial differences. The trend of regeneration performance was MoO_3_ > V_2_O_5_ > Cr_2_O_3_ > TiO_2_ > WO_3_ > blank test.

Loading metal oxides onto catalyst supports enhances catalytic activity by increasing the acid sites of the catalyst. Zhang et al. [7] prepared a composite catalyst, SO_4_^2−^/ZrO_2_/γ-Al_2_O_3_ (SZA), with varying mass ratios of ZrO_2_ and γ-Al_2_O_3_, and employed it for the first time in the mono-ethanolamine (MEA) rich solution regeneration process. The results indicated that the SZA catalyst exhibited superior catalytic activity compared to the precursor catalyst. Zhang et al. [8] prepared a series of bifunctional Al_2_O_3_/H-ZSM-5 catalysts (Al-ZSM) using the ultrasonic precipitation method for the first time and utilized them in the CO_2_ desorption process. The regeneration behavior of four Al-ZSM catalysts for 5M MEA solvent was studied under the conditions of an initial CO_2_ loading of 0.5 mol CO_2_/mol amine and a temperature of 96 °C. The results revealed that all catalysts improved the CO_2_ desorption performance, with the Al-ZSM catalyst exhibiting higher catalytic performance than single catalyst Al_2_O_3_ and H-ZSM-5. In comparison to the test without a catalyst, Al-ZSM reduced the heat load by 23.3–34.2%. When Al-ZSM was employed for MEA regeneration, the desorption performance was 2–3 times higher than that of the blank process. Bhatti et al. [9] synthesized inexpensive M-montmorillonite (M = Cr, Fe, Co) catalysts through a straightforward metal ion exchange process and employed them to optimize the CO_2_ desorption rate of 30 wt% MEA solution at medium temperature (≤86 °C). The results indicated that, compared to the non-catalytic MEA solution, the CO_2_ desorption rate and CO_2_ desorption capacity increased by 315% and 82.5%, respectively, and the regeneration energy consumption decreased by 40%. Wei et al. [10] introduced heteropoly acids into cerium-based MOFs to increase acidic sites. The results demonstrated that the composite catalyst CeO_2_-MOFHPW (CeM-HPW) exhibited robust catalytic performance. In comparison to the non-catalytic process, the CO_2_ desorption capacity and desorption rate increased by 38.1% and 166%, respectively, and the desorption energy consumption decreased by 29.4%. Hetero-polyacid anions play a crucial role in the deprotonation process of proton transfer. The catalytic pathway lowers the energy barrier of the desorption reaction, thereby achieving an efficient and low-energy desorption effect.

γ-Al_2_O_3_ possesses several advantageous properties that make it an ideal catalyst support material. It exhibits a large specific surface area, providing numerous active sites for catalytic reactions. Moreover, it demonstrates exceptional thermal stability, making it suitable for high-temperature catalyst applications. Its high porosity, characterized by microscopic pores, further enhances mass transfer efficiency and facilitates the availability of additional active sites. Additionally, γ-Al_2_O_3_ exhibits commendable chemical stability, enabling it to maintain catalytic activity even under harsh operating conditions [11,12,13,14]. Furthermore, in various other domains, metal-supported catalysts have demonstrated excellent catalytic performance [15,16,17]. Consequently, the utilization of a metal oxide-supported γ-Al_2_O_3_ catalyst holds great potential for facilitating carbon dioxide desorption and reducing regeneration energy consumption.

Mao et al. [18] investigated the absorption and desorption characteristics of the BZA-AEP mixed amine absorbent, which demonstrated remarkable performance. In comparison to mono-ethanolamine (MEA), the BZA-AEP absorbent exhibited a 48% increase in average CO_2_ absorption rate, a 120% enhancement in CO_2_ desorption capacity, and a 161% rise in average CO_2_ desorption rate. However, the regeneration efficiency of BZA-AEP was approximately 55%. To further augment its desorption efficacy, two Lewis acid catalysts were synthesized in this study, and the catalytic desorption properties of these catalysts for BZA-AEP were examined.

## 2. Results and Discussion

### 2.1. Catalytic Desorption Performance of CeO_2_-γ-Al_2_O_3_

**Figure 1:**
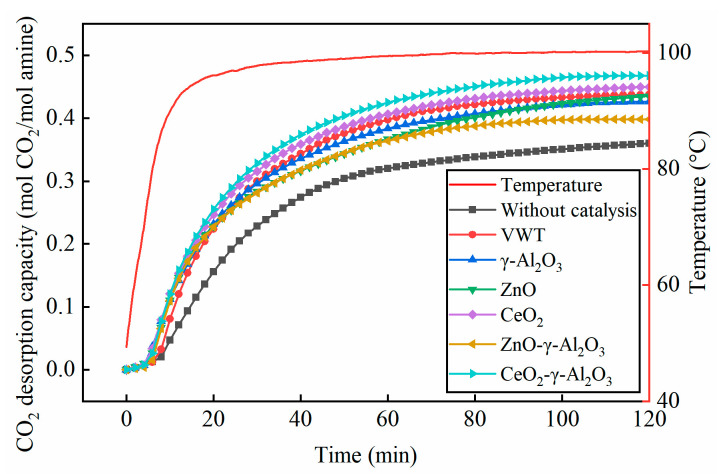
The change of desorption capacity of BZA-AEP desorbed for 2 h under the action of catalyst.

The desorption capacity of BZA-AEP was investigated for 2 h using various catalysts, including a commercial VWT catalyst, three precursor catalysts, and two M-γ-Al_2_O_3_ (M = ZnO or CeO_2_) Lewis acid catalysts. It can be seen from Figure 1 that CeO_2_-γ-Al_2_O_3_ exhibited the best catalytic effect, and the capacity of carbon dioxide desorption increased by 30% compared with the case without catalyst. The characterization analysis showed that this benefited from the large specific surface area and acidity of CeO_2_-γ-Al_2_O_3_, which increased active catalytic surface. The catalyzed desorption performance follows the order: CeO_2_-γ-Al_2_O_3_ > CeO_2_ > VWT ≈ ZnO > γ-Al_2_O_3_ > ZnO-γ-Al_2_O_3_ > without catalyst. During the initial 20 min, the desorption capacity for each catalyst in the high carbon dioxide-loaded absorbent exhibit minimal differences, indicating that each catalyst possesses a strong catalytic desorption capability. In the mechanism of Lewis acid catalyst catalysis [3], the acidic sites on the catalyst’s surface interact with oxygen atoms in the absorbent, facilitating the decomposition of carbamate and the subsequent release of carbon dioxide.

**Figure 2:**
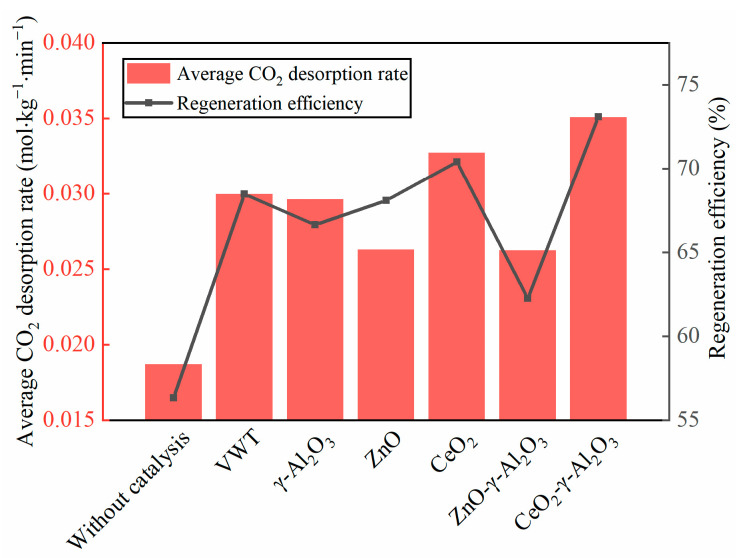
The average CO_2_ desorption rate and regeneration efficiency of BZA-AEP under the action of catalyst.

Figure 2 compares the average CO_2_ desorption rate and regeneration efficiency of BZA-AEP catalyzed by the no catalyst, commercial VWT catalyst, three precursor catalysts and two M-γ-Al_2_O_3_ Lewis acid catalysts. The results indicate that the CeO_2_-γ-Al_2_O_3_ Lewis acid catalyst outperforms the others, exhibiting superior average CO_2_ desorption rates and regeneration efficiencies. As seen in Figure 2, the average CO_2_ desorption rate order is as follows: CeO_2_-γ-Al_2_O_3_ > CeO_2_ > VWT > γ-Al_2_O_3_ > ZnO ≈ ZnO-γ-Al_2_O_3_ > without catalyst. The CeO_2_-γ-Al_2_O_3_ Lewis acid catalyst increases the average CO_2_ desorption rate of BZA-AEP by 87% compared to the no catalyst and by 17% compared to the commercial VWT catalyst, highlighting its exceptional performance in enhancing desorption rates. In terms of regeneration efficiency, the catalyst order is as follows: CeO_2_-γ-Al_2_O_3_ > CeO_2_ > VWT ≈ ZnO > γ-Al_2_O_3_ > ZnO-γ-Al_2_O_3_ > without catalyst. Under the catalysis of the CeO_2_-γ-Al_2_O_3_ Lewis acid catalyst, the regeneration efficiency of BZA-AEP reaches 73%, which is 30% and 7% higher than that of the no catalyst and the commercial VWT catalyst, respectively. This further substantiates the superior performance of the CeO_2_-γ-Al_2_O_3_ Lewis acid catalyst in promoting BZA-AEP regeneration.

### 2.2. Effect of Temperature on Catalytic Desorption Performance of CeO_2_-γ-Al_2_O_3_

**Figure 3:**
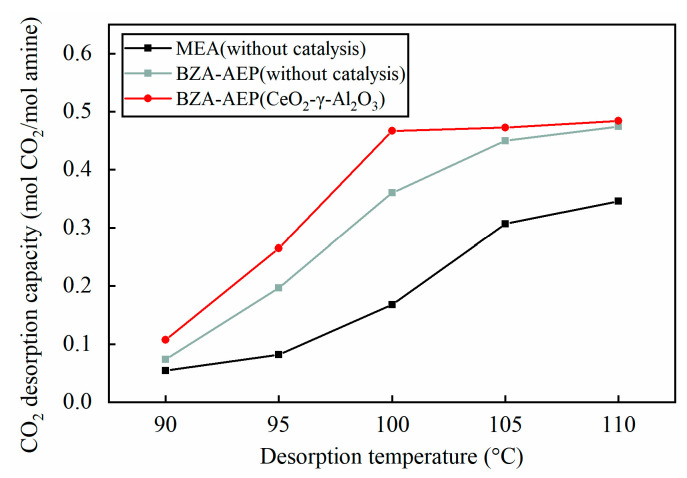
Relationship between desorption temperature and desorption capacity under CeO_2_-γ-Al_2_O_3_ catalysis.

The impact of CeO_2_-γ-Al_2_O_3_ catalyst on the catalytic desorption of BZA-AEP was investigated for a duration of 2 h at desorption temperatures ranging from 90 °C to 110 °C. It can be clearly seen from Figure 3 that, with the increase of the desorption temperature, the difference in the desorption capacity within 2 h between catalyst catalysis and no catalyst catalysis presents a first increasing and then decreasing trend. Notably, the CeO_2_-γ-Al_2_O_3_ catalyst demonstrates the most significant increase in BZA-AEP desorption capacity at a 100 °C desorption temperature. Under the catalysis of CeO_2_-γ-Al_2_O_3_, the desorption capacity of BZA-AEP increased by 2–46% compared to no catalyst, signifying the substantial advantage of the CeO_2_-γ-Al_2_O_3_ catalyst in improving BZA-AEP desorption capacity. Furthermore, in comparison with the MEA desorption capacity of no catalyst, the desorption amount of BZA-AEP catalyzed by CeO_2_-γ-Al_2_O_3_ increased by 40–222%.

**Figure 4:**
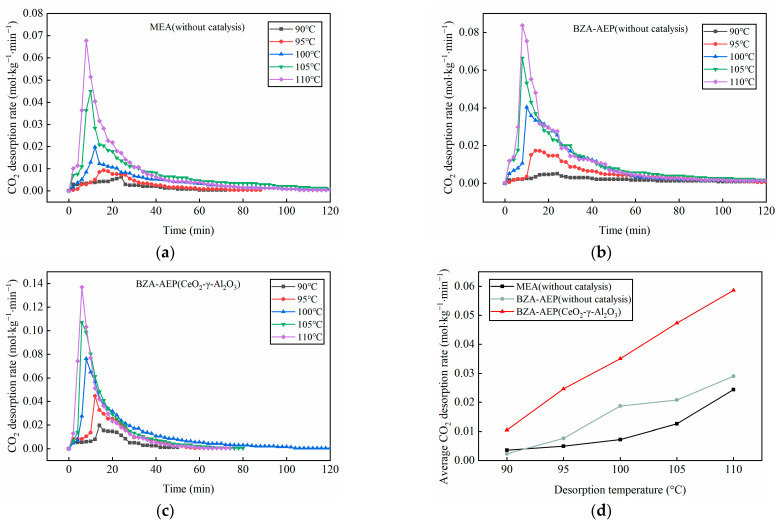
Relationship between desorption temperature and carbon dioxide desorption rate under CeO_2_-γ-Al_2_O_3_ catalysis. (**a**) MEA(Non-catalytic), (**b**) BZA-AEP(Non-catalytic), (**c**) BZA-AEP(CeO_2_-γ-Al_2_O_3_) and (**d**) the effect of desorption temperature on the average CO_2_ desorption rate.

As depicted in Figure 4c, the maximum desorption rate of BZA-AEP under CeO_2_-γ-Al_2_O_3_ catalysis increased with rising desorption temperature, and the time required to reach the maximum desorption rate gradually shortened. Figure 4a–c show that CeO_2_-γ-Al_2_O_3_ exhibits a superior catalytic effect at desorption temperatures of 90 °C, 95 °C, 100 °C, 105 °C and 110 °C, making the maximum CO_2_ desorption rate of BZA-AEP, respectively, increased by 292%, 159%, 89%, 61% and 64% and, compared with MEA without catalysis, increased by 211%, 380%, 286%, 138% and 102%, respectively. Figure 4d reveals that the average CO_2_ desorption rate linearly increases with desorption temperature. The CeO_2_-γ-Al_2_O_3_ catalyst can reduce the desorption temperature of BZA-AEP from 110 °C to 100 °C. Under CeO_2_-γ-Al_2_O_3_ catalysis, the average desorption rate of BZA-AEP surpasses that of BZA-AEP without catalysis by 87–354%, and exceeds that of MEA without catalysis by 141–400%. These data conclusively confirm that the CeO_2_-γ-Al_2_O_3_ Lewis acid catalyst significantly increases the CO_2_ desorption rate of BZA-AEP at various desorption temperatures.

### 2.3. Cyclic Catalytic Desorption Performance of CeO_2_-γ-Al_2_O_3_

**Figure 5:**
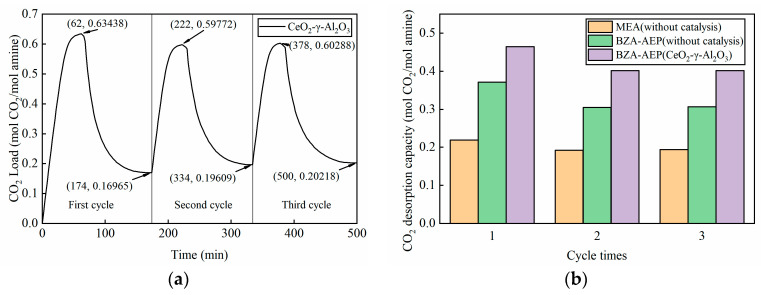
BZA-AEP (CeO_2_-γ-Al_2_O_3_) cycle absorption and desorption performance. (**a**) CO_2_ load change and (**b**) desorption capacity change.

According to the results in Section 3.2, the cycle desorption temperature was set at 100 °C. As observed in Figure 5a, the loading of the BZA-AEP rich solution reached 0.63438 mol CO_2_/mol amine during the initial absorption and desorption process. In the subsequent two cycles, the rich solution load experienced a slight decrease, stabilizing at approximately 0.6 mol CO_2_/mol amine. This decrease can be attributed to some amine absorbent and carbamates were adsorbed on the surface of the fresh catalyst during the first cycle, so that this part of amine absorbent failed to enter the absorber to participate in the absorption process. Concurrently, the lean liquid loading increased marginally in the last two cycles compared to the first cycle and stabilized at around 0.2 mol CO_2_/mol amine, further corroborating the hypothesis that some amine absorbent and carbamates were adsorbed on the catalyst surface. It can be seen from Figure 5b that the desorption capacity of the absorbent tends to be stable after the first cycle. Under the catalysis of CeO_2_-γ-Al_2_O_3_, the cycle capacity of BZA-AEP reaches 0.40147 mol CO_2_/mol amine, which is 31% higher than that of uncatalyzed BZA-AEP and 108% higher than that of uncatalyzed MEA.

**Figure 6:**
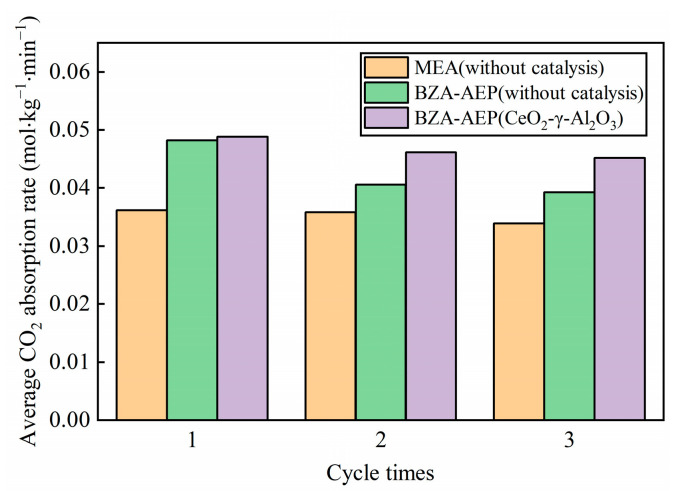
The relationship between cycle times and the average CO_2_ absorption rate for BZA-AEP (CeO_2_-γ-Al_2_O_3_).

It can be seen from Figure 6 that the average absorption rate of BZA-AEP catalyzed by CeO_2_-γ-Al_2_O_3_ is higher than that of uncatalyzed BZA-AEP during the second and third cycles. The reason for this phenomenon is that the BZA-AEP catalyzed by CeO_2_-γ-Al_2_O_3_ releases more carbon dioxide during the desorption process. The concentration of amines not bound to carbon dioxide in BZA-AEP lean solution catalyzed by CeO_2_-γ-Al_2_O_3_ is higher than that in BZA-AEP lean solution without catalysis: the higher the amine concentration, the faster the reaction rate of amine and carbon dioxide.

**Figure 7:**
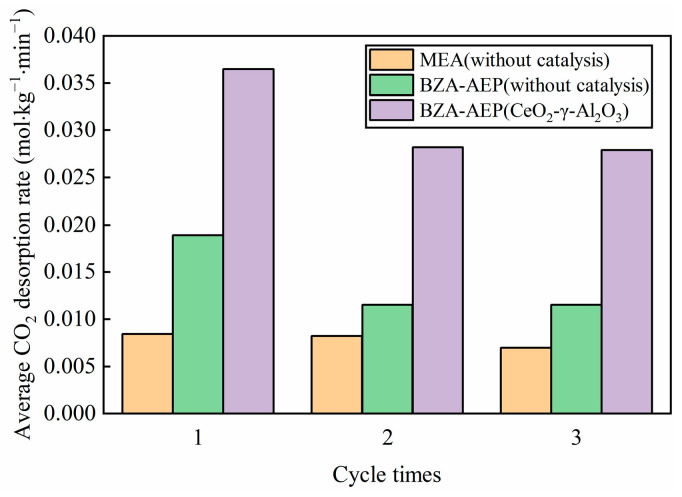
The relationship between cycle times and the average CO_2_ desorption rate for BZA-AEP (CeO_2_-γ-Al_2_O_3_).

From Figure 7, we can observe that the average CO_2_ desorption rate of BZA-AEP catalyzed by CeO_2_-γ-Al_2_O_3_ starts to stabilize after the second cycle, indicating the catalyst’s good stability. Among them, the average CO_2_ desorption rate of the second cycle is lower than that of the first cycle due to a decrease in the rich solution load caused by the exclusion of a portion of the amine solution from the absorption cycle. Consequently, the desorption capacity and average CO_2_ desorption rate decrease accordingly. Compared with the uncatalyzed BZA-AEP, the average CO_2_ desorption rate of BZA-AEP catalyzed by CeO_2_-γ-Al_2_O_3_ increased by 144%. Compared with uncatalyzed MEA, the average CO_2_ desorption rate of BZA-AEP catalyzed by CeO_2_-γ-Al_2_O_3_ increased by 268%.

### 2.4. Characterization of CeO_2_-γ-Al_2_O_3_ Catalyst

#### 2.4.1. SEM Characterization

**Figure 8:**
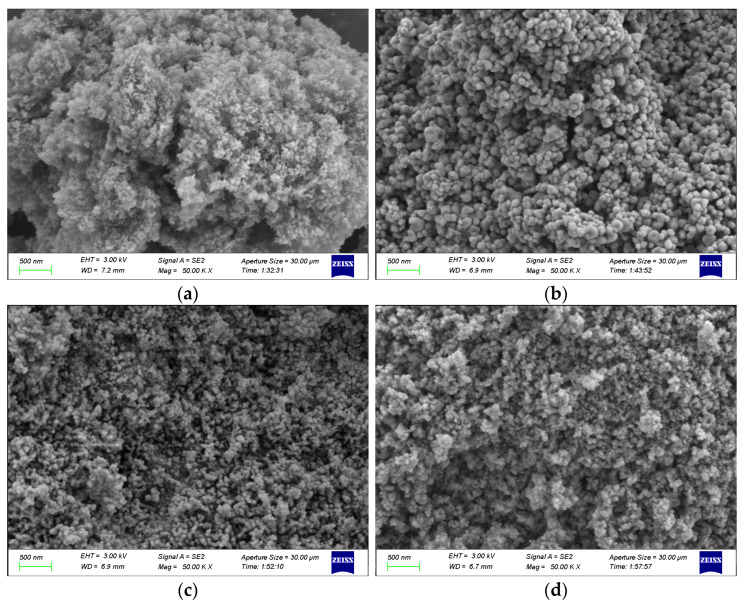
SEM images of catalysts. (**a**) γ-Al_2_O_3_, (**b**) CeO_2_, (**c**) CeO_2_-γ-Al_2_O_3_ and (**d**) CeO_2_-γ-Al_2_O_3_ after three times cycle.

**Figure 9:**
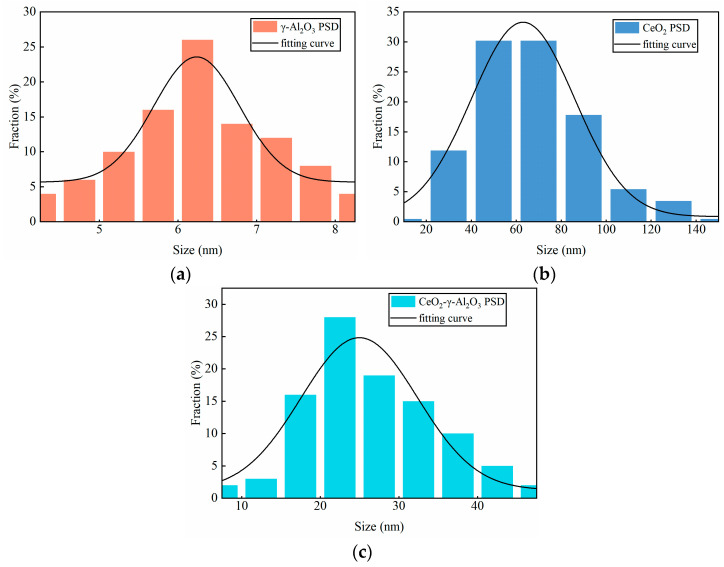
Catalyst particle size distribution (**a**) γ-Al_2_O_3_, (**b**) CeO_2_ and (**c**) CeO_2_-γ-Al_2_O_3_.

The morphology of the catalyst was examined utilizing the Sigma 300 scanning electron microscope (SEM) both before and after its usage. Figure 8a–d display the SEM images of γ-Al_2_O_3_, CeO_2_, fresh CeO_2_-γ-Al_2_O_3_, and CeO_2_-γ-Al_2_O_3_ catalyst after three times cycle, respectively. The γ-Al_2_O_3_ (Figure 8a) shows that the catalyst is aggregated from extremely fine particles. The SEM images of CeO_2_ (Figure 8b) corroborate the spherical morphology of CeO_2_ nanoparticles [19]. The fresh CeO_2_-γ-Al_2_O_3_ catalyst (Figure 8c) displays a high dispersion of γ-Al_2_O_3_ among CeO_2_ nanoparticles. In the SEM image of the CeO_2_-γ-Al_2_O_3_ catalyst (Figure 8d) after three times cycle, we can see that the surface of the catalyst has not changed significantly, but the aggregation between the particles has become tighter. This observation aligns with the experimental data presented in previous sections, further substantiating the exceptional stability of the CeO_2_-γ-Al_2_O_3_ catalyst. Figure 9 reveals that the catalyst particle size distribution follows a Gaussian distribution. The γ-Al_2_O_3_ catalyst has a mean particle size of 6.35 nm, with a standard deviation of 1.06 nm. The CeO_2_ catalyst exhibited an average particle size of 67.17 nm and a standard deviation of 24.59 nm. The CeO_2_-γ-Al_2_O_3_ catalyst displayed an average particle size of 26.76 nm and a standard deviation of 8.40 nm.

#### 2.4.2. XRD Characterization

To determine any changes in the structure of the catalyst, both before and after use, the Ultima IV X-ray powder diffractometer was employed. Prior to the test, the catalyst was dried and pressed. The diffraction patterns were obtained using continuous scanning with a range of 10–80° (2θ) and a scanning rate of 5°/min.

**Figure 10:**
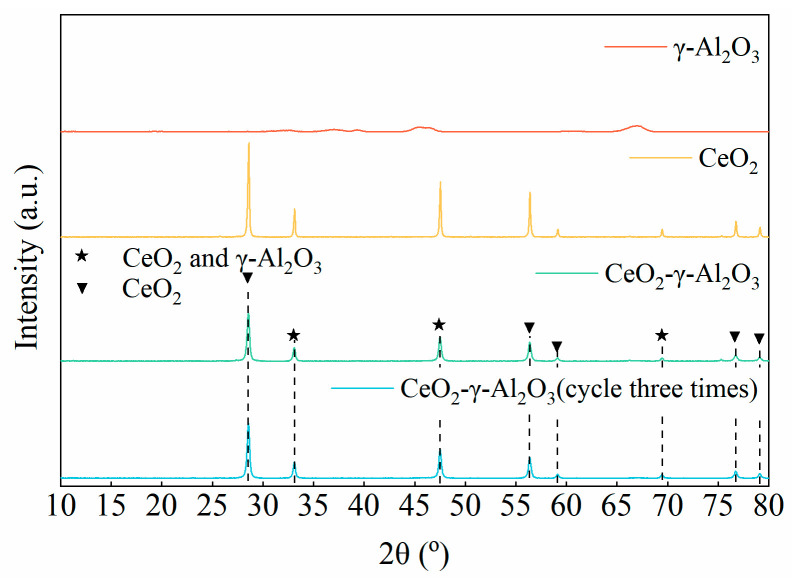
Catalyst XRD diffraction pattern.

Figure 10 displays the crystal structure diffraction patterns of the two precursor catalysts (γ-Al_2_O_3_ and CeO_2_), the fresh CeO_2_-γ-Al_2_O_3_ catalyst, and the CeO_2_-γ-Al_2_O_3_ catalyst after three times cycle. The diffraction pattern of γ-Al_2_O_3_ exhibits a weak characteristic peak intensity, indicating a small grain size. Moreover, only the diffraction peak of CeO_2_ can be observed on the CeO_2_-γ-Al_2_O_3_ catalyst, with no diffraction peak of crystalline γ-Al_2_O_3_ evident. This suggests that γ-Al_2_O_3_ may be highly dispersed on CeO_2_ or present as clusters, surpassing the XRD detection limit [20,21,22,23,24]. The crystal structures of γ-Al_2_O_3_ (PDF-ICDD 01-079-1558) [25], CeO_2_ (PDF-ICDD 00-043-1002) [26], and CeO_2_-γ-Al_2_O_3_ belong to the cubic system. The characteristic peaks of the CeO_2_-γ-Al_2_O_3_ catalyst before and after cycling remain essentially unchanged, indicating that the cycling process does not impact the catalyst’s structure. The diffraction peak intensity varies among these catalysts. A narrower peak corresponds to a larger grain size and better crystallinity, while a wider peak signifies a smaller grain size and poorer crystallinity [27,28,29]. The CeO_2_-γ-Al_2_O_3_ catalyst possesses a highly ordered crystal structure, distinct diffraction peaks, and narrow peak width, which implies good crystallinity and high stability—crucial for long-term catalytic applications.

Table 1 presents the grain sizes of γ-Al_2_O_3_, CeO_2_, CeO_2_-γ-Al_2_O_3_, and CeO_2_-γ-Al_2_O_3_ after three times cycle, as determined using the Debye-Scherrer equation [30,31] (Dβ = Kλ/βcosθ), based on the strongest diffraction peak. Additionally, the interplanar spacing of the strongest peaks of these catalysts is computed using the Bragg equation [32] (2dsinθ = nλ). The CeO_2_-γ-Al_2_O_3_ catalyst exhibits a smaller grain size compared to the CeO_2_ catalyst, resulting in a larger active surface during the catalytic reaction. The addition of CeO_2_ to γ-Al_2_O_3_ also enhances its thermal stability and mitigates the sintering of CeO_2_ nanoparticles [33], thereby maintaining the catalyst’s long-term activity. Given that the ionic radius of Al^3+^ (0.54 Å) is smaller than that of Ce^4+^ (0.92 Å), the lattice constant of the CeO_2_-γ-Al_2_O_3_ catalyst is slightly reduced compared to the CeO_2_ catalyst. This observation indicates that some Al^3+^ ions may be doped into the surface lattice of the CeO_2_ catalyst. Such doping aids in enhancing the stability and catalytic activity of the catalyst, allowing the CeO_2_-γ-Al_2_O_3_ catalyst to exhibit superior performance during the desorption process.

#### 2.4.3. BET and NH_3_-TPD Characterization

The specific surface area of the catalyst was measured using the fully automatic surface area and porosity analysis of the ASAP 2460. Prior to testing the catalyst, a vacuum degassing pre-treatment was carried out at a temperature of 200 °C for 4 h. The BET (Brunauer Emmett Teller) method was used to calculate the specific surface area of the catalyst. To examine the concentration distribution of catalyst acid, including TCD detector, the AutoChem II 2920 chemisorption instrument was utilized. Before the sample test, the temperature was raised to 400 °C at a rate of 10 °C/min in an argon atmosphere and maintained for 1 h to eliminate any physically adsorbed water and impurities from the sample surface. The temperature was then reduced to 50 °C. Next, a 10% NH_3_-He gas flow was introduced onto the catalyst surface for adsorption saturation, followed by a high-purity He gas blow for 1 h to remove any weak physical adsorption of NH_3_ on the surface. Finally, the NH_3_-TPD curve was obtained by heating up to 450 °C at a rate of 10 °C/min. The Gaussian deconvolution method was applied to perform a semi-quantitative analysis of the TPD curve to determine the acidity of the catalyst.

**Figure 11:**
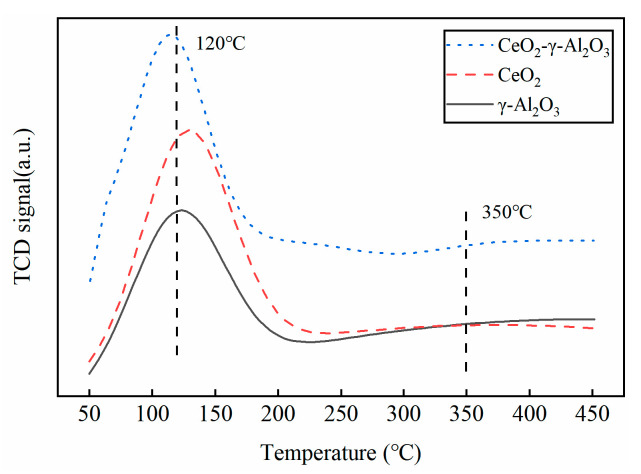
Acid strength distribution of CeO_2_-γ-Al_2_O_3_ catalyst.

Table 2 reveals the variations in specific surface area and acid strength among the three catalysts. γ-Al_2_O_3_ exhibits a significantly larger specific surface area, suggesting the availability of a greater number of active surfaces for catalytic reactions. In contrast, the specific surface area of CeO_2_ is relatively small, measuring only 2.3596 m^2^/g. Loading CeO_2_ onto γ-Al_2_O_3_ leads to an increase in the specific surface area of CeO_2_, but simultaneously reduces the specific surface area of γ-Al_2_O_3_. Figure 11 displays the catalyst’s acid strength distribution characterized by NH_3_-TPD. The NH_3_ desorption peak at 100–200 °C typically corresponds to the weak acid site, the NH_3_ desorption peak in the range of 200–400 °C corresponds to the medium acid site, and the NH_3_ desorption peak above 400 °C corresponds to the strong acid site [34]. As evident in Figure 11, the low-temperature desorption peak near 120 °C arises from the catalyst’s weak acid site, while the medium-temperature desorption peak near 350 °C is attributable to the catalyst’s medium-strong acid site. Table 2 shows that the weak acid sites of the CeO_2_-γ-Al_2_O_3_ catalyst are significantly stronger than those of the two parent catalysts. The weak acid sites of the CeO_2_-γ-Al_2_O_3_ catalyst increase by 287% and 20% compared to γ-Al_2_O_3_ and CeO_2_ parent catalysts, respectively. This suggests that CeO_2_ loading enhances the weak acid sites of the γ-Al_2_O_3_ catalyst.

### 2.5. Catalytic Mechanism of CeO_2_-γ-Al_2_O_3_

According to the zwitterionic mechanism and the alkali-catalyzed bicarbonate reaction mechanism, the regeneration process of primary and secondary amines comprises two distinct steps: cleavage of the N-C bond of carbamates and deprotonation of protonated amines [35]. On the other hand, the regeneration process of tertiary amines involves bicarbonate hydrogenation decomposition and protonated amine deprotonation [36]. However, previous studies have indicated that CO_2_ desorption can be accelerated by providing a substantial number of Brønsted acid sites, Lewis acid sites, and HCO_3_^−^-like alkaline groups [37,38].

**Figure 12:**
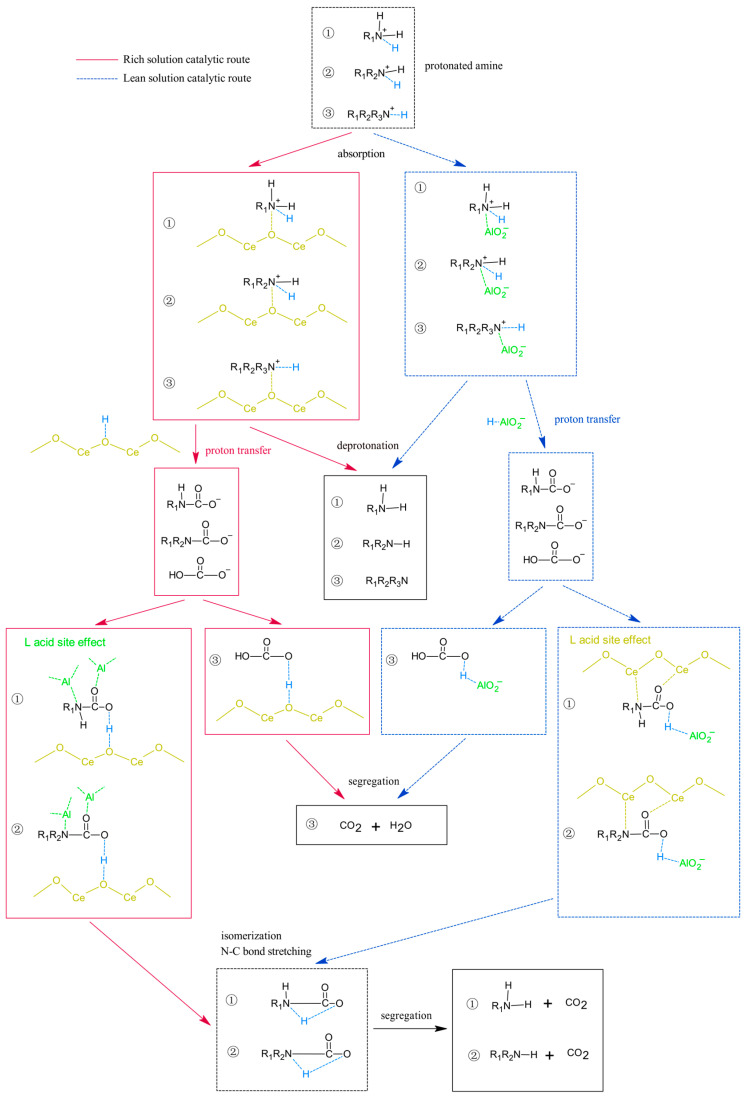
Mechanism of CeO_2_-γ-Al_2_O_3_ catalyst for CO_2_ desorption.

Based on the above perspective, Figure 12 depicts the mechanism diagram of CO_2_ desorption catalyzed by CeO_2_-γ-Al_2_O_3_. The figure illustrates three different catalytic desorption processes for various absorbents (① for primary amine, ② for secondary amine, and ③ for tertiary amine). During the desorption process, the absorbent transitions from a rich solution to a lean solution, resulting in an increase in the pH value of the solution. Additionally, the catalytic desorption pathways vary in alkaline environments.

The rich solution primarily undergoes the following catalytic desorption process. Due to weak alkalinity, the AlO_2_^−^ anion cannot form in the rich solution region [31], while the oxygen atom of CeO_2_ readily receives H^+^, as it is more electronegative than the nitrogen atom on the amino group [10]. Consequently, the rich solution region is mainly CeO_2_ to promote the deprotonation of protonated amine. The positively charged protonated amine (N atom) first adsorbs on the more negatively charged CeO_2_ basic site (O atom) according to the principle of opposites attract, and the proton is transferred from the nitrogen atom of the amino group to the oxygen atom of CeO_2_, completing the deprotonation. Subsequently, CeO_2_ transfers surface protons to carbamates and bicarbonates. Carbamates acquire protons, and the Lewis acid sites on the γ-Al_2_O_3_ surface attack the O and N atoms of carbamates [3], promoting the stretching of N-C bonds and weakening bond strength, thus reducing the activation energy of the carbamate fracture reaction. Finally, through isomerization [4], the carbamate’s proton transfers from the O atom to the nearby N atom, breaking the N-C bond and decomposing into amines and CO_2_. Bicarbonate directly decomposes into H_2_O and CO_2_ after obtaining the protons transferred from CeO_2_.

The lean solution primarily undergoes the following catalytic desorption process. In the strong alkaline environment of the lean solution, the surface of Al_2_O_3_ exhibits electronegativity and forms an AlO_2_^−^ basic group, as demonstrated by previous studies [38]. These anions capture protons from protonated amines and subsequently transfer them to carbamates via water. This process predominantly occurs in the lean solution region, meaning that, as CO_2_ progressively desorbs, the absorbent’s alkalinity gradually increases. The electronegativity of the oxygen atom for CeO_2_ is less than that of the AlO_2_^−^ basic group. hence, the AlO_2_^−^ basic group is primarily responsible for proton transfer from the protonated amine in the lean solution region. The AlO_2_^−^ basic group first transfers protons from the protonated amine to carbamate and bicarbonate. Then, due to the hole donor nature of CeO_2_ [20], the Lewis acid site of CeO_2_ can bind to the electron pair donor, allowing carbamate to accept the proton and be attacked by the Lewis acid site on the CeO_2_ surface at its O and N atoms. This process results in the stretching of the N-C bond and the weakening of the bond energy, thereby reducing the activation energy of the carbamate cleavage reaction. Ultimately, through isomerization [4], the N-C bond of carbamate is broken, and the compound decomposes into amines and CO_2_. Simultaneously, bicarbonate directly decomposes into H_2_O and CO_2_ after obtaining the proton transferred from the AlO_2_^−^ basic group.

## 3. Materials and Methods

### 3.1. Catalyst Preparation Materials and Methods

**Figure 13:**
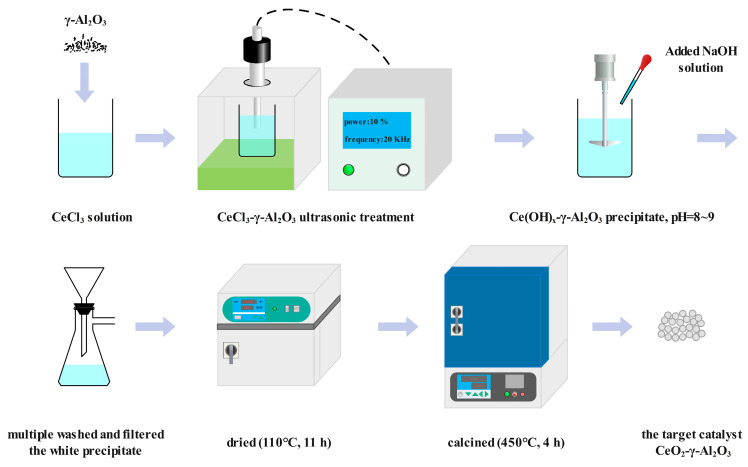
Ultrasound-assisted precipitation catalyst synthesis pathway.

The chemical reagents used in the experiment are shown in Table 3. The catalyst was synthesized using an ultrasonic-assisted precipitation method. Following the reported synthesis method [8], the specific synthesis pathway is depicted in Figure 13. Initially, CeCl_3_·6H_2_O was added to 500 mL of deionized water, resulting in an aqueous solution of CeCl_3_. Subsequently, a suitable amount of γ-Al_2_O_3_ powder was added to the solution, forming a suspension with a CeO_2_ to γ-Al_2_O_3_ molar ratio of 1. Ultrasonic treatment was applied to the suspension at room temperature, using an ultrasonic disperser operating at 10% power and 20 kHz for 0.5 h to ensure comprehensive mixing of the constituents. Next, a NaOH solution was gradually added to the suspension at room temperature with continuous stirring until the pH reached approximately 8–9. The system was then allowed to stand at room temperature for 2 h, resulting in the formation of a precipitate. The precipitate was subsequently washed with deionized water and filtered multiple times. The filtered precipitate was dried at 110 °C for 11 h in a blast drying oven. To obtain the desired catalyst CeO_2_-γ-Al_2_O_3_, the dried solid was calcined in a muffle furnace at 800 °C for 4 h. Employing the same preparation method, the ZnO-γ-Al_2_O_3_ solid acid catalyst was obtained.

### 3.2. Desorption Experimental Materials and Steup

**Figure 14:**
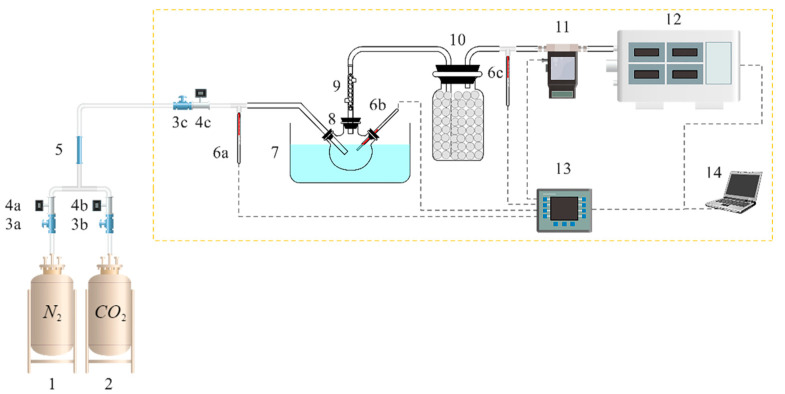
Flow chart of the experimental setup [11] (1. N_2_ cylinder, 2. CO_2_ cylinder, 3. flow control valve (3a,3b,3c), 4. gas flow meter (4a,4b,4c), 5. gas mixer, 6. temperature sensor (6a,6b,6c), 7. oil bath, 8. three-neck flask, 9. condenser tube, 10. drying bottle, 11. mass flow meter, 12. flue gas analyzer 13. data acquisition instrument, 14. computer).

The chemical compounds utilized in this study, namely ethanolamine (MEA, 99%), benzylamine (BZA, 99%), aminoethylpiperazine (AEP, 99%), carbon dioxide (CO_2_, 99.9%), and nitrogen (N_2_, 99.9%), were acquired without further purification. The specific conditions of the desorption experiment are presented in Table 4, and the experiment was executed utilizing the absorption and desorption experimental apparatus, as described in reference [11]. The specific experimental setup is shown in Figure 14. Prior to commencing the experiment, the system underwent a leak test and N_2_ purge. For the absorption experiments, valves 3a, 3b, and 3c were opened, and the inlet CO_2_ concentration was set to 5% with an inlet gas flow of 1.25 L/min, an absorption temperature of 50 °C, and an absorption solution of 50 g. For the desorption experiments, valves 3a, 3b, and 3c were closed, and the desorption temperature was set to 100 °C. Each group of experiments was repeated three times, and the results were averaged.

## 4. Conclusions

1. Through a comparison of the catalytic desorption performance of two Lewis acid catalysts, three precursor catalysts, and one commercial catalyst (VWT), CeO_2_-γ-Al_2_O_3_ exhibited superior catalytic desorption effect over other catalysts. In comparison to no catalyst, CeO_2_-γ-Al_2_O_3_ Lewis acid catalyst increased the desorption capacity of BZA-AEP by 30%, the average desorption rate by 87%, and the regeneration efficiency by 30%.
2. The catalytic desorption effect of CeO_2_-γ-Al_2_O_3_ is particularly significant at low temperatures, reducing the desorption temperature of BZA-AEP by approximately 10 °C.
3. CeO_2_-γ-Al_2_O_3_ cyclic catalytic desorption results show that CeO_2_-γ-Al_2_O_3_ has good catalytic stability. At the same time, through the characterization of the surface structure and crystal structure of the catalyst, there is no change in the structure of the catalyst before and after cycling.
4. The catalytic mechanism of CeO_2_-γ-Al_2_O_3_ varies in different alkaline environments. The interaction between CeO_2_ and γ-Al_2_O_3_ provides the catalyst with a strong catalytic effect in both rich and poor solution.

## Figures and Tables

**Table 1:** Crystal parameters of the catalyst.

Catalyst	Grain Size (nm)	2θ (°)	Crystal Face (hkl)	Crystal Plane Spacing (Å)
γ-Al_2_O_3_	5.4	67.0543	440	1.3946
CeO_2_	46.3	28.5500	111	3.1240
γ-Al_2_O_3_-CeO_2_	25.6	28.5718	111	3.1216
γ-Al_2_O_3_-CeO_2_ (after three times cycle)	26.0	28.6107	111	3.1175

**Table 2:** Specific surface area and acid strength of CeO_2_-γ-Al_2_O_3_ catalyst.

Catalyst	Specific Surface Area (m^2^/g)	Acid Strength (mmol/g)	
		Weak Acid	Total Acid
γ-Al_2_O_3_	149.3333	0.412	1.485
CeO_2_	2.3596	1.332	1.801
CeO_2_-γ-Al_2_O_3_	36.8573	1.594	2.098

**Table 3:** Catalyst preparation materials.

Reagent Name	Abbreviation	Specification
deionized water	DI	-
zinc sulfate heptahydrate	ZnSO_4_·7H_2_O	99.5%
cerium chloride hexahydrate	CeCl_3_·6H_2_O	99.99%
sodium hydroxide	NaOH	95%
gamma alumina	γ-Al_2_O_3_	99.99%
carbon dioxide	CO_2_	99.9%
nitrogen	N_2_	99.9%
V_2_O_5_-WO_3_/TiO_2_	VWT	TiO_2_:V_2_O_5_:WO_3_:SiO_2_ = 86:8.2:4:1

**Table 4:** Desorption experimental conditions.

Name	Parameter
absorbent	BZA-AEP
total amine concentration	3 mol/kg
concentration ratio of BZA to AEP	1.5
amount of absorbent	50 g
absorbent rich solution	Saturated solution under 5% CO_2_ + 95% N_2_
amount of catalyst	3 g

## Data Availability

Not applicable.
